# *iso*‐BAI Guided Surface Recrystallization for Over 14% Tin Halide Perovskite Solar Cells

**DOI:** 10.1002/advs.202309668

**Published:** 2024-03-27

**Authors:** Pok Fung Chan, Minchao Qin, Chun‐Jen Su, Liping Ye, Xuezhou Wang, Yunfan Wang, Xin Guan, Zhen Lu, Gang Li, To Ngai, Sai Wing Tsang, Ni Zhao, Xinhui Lu

**Affiliations:** ^1^ Department of Physics The Chinese University of Hong Kong New Territories Hong Kong SAR 999077 China; ^2^ National Synchrotron Radiation Research Center Hsinchu Science Park Hsinchu 30076 Taiwan; ^3^ Department of Chemistry The Chinese University of Hong Kong New Territories Hong Kong SAR 999077 China; ^4^ Department of Electronic Engineering The Chinese University of Hong Kong New Territories Hong Kong SAR 999077 China; ^5^ Department of Materials Science and Engineering City University of Hong Kong Kowloon Tong Hong Kong SAR 999077 China; ^6^ Department of Electrical and Electronic Engineering The Hong Kong Polytechnic University Hung Hom Hong Kong SAR 999077 China

**Keywords:** crystallization, lead‐free perovskites, surface treatment, tin‐halide perovskites, tin oxidation

## Abstract

Tin‐based perovskite solar cells (PSCs) are promising environmentally friendly alternatives to their lead‐based counterparts, yet they currently suffer from much lower device performance. Due to variations in the chemical properties of lead (II) and tin (II) ions, similar treatments may yield distinct effects resulting from differences in underlying mechanisms. In this work, a surface treatment on tin‐based perovskite is conducted with a commonly employed ligand, iso‐butylammonium iodide (*iso*‐BAI). Unlike the passivation effects previously observed in lead‐based perovskites, such treatment leads to the recrystallization of the surface, driven by the higher solubility of tin‐based perovskite in common solvents. By carefully designing the solvent composition, the perovskite surface is effectively modified while preserving the integrity of the bulk. The treatment led to enhanced surface crystallinity, reduced surface strain and defects, and improved charge transport. Consequently, the best‐performing power conversion efficiency of FASnI_3_ PSCs increases from 11.8% to 14.2%. This work not only distinguishes the mechanism of surface treatments in tin‐based perovskites from that of lead‐based counterparts, but also underscores the critical role in designing tailor‐made strategies for fabricating efficient tin‐based PSCs.

## Graphical abstract

The surface recrystallization triggered by *iso*‐BAI is identified as a distinct mechanism in tin‐based perovskites, compared to the lead‐based counterparts. GIWAXS results suggest that the defective tin perovskite surfaces are dissolved by the moderate solvent, followed by a guided recrystallization with the assistance of *iso*‐BAI, resulting in a promising power conversion efficiency of 14.2%.

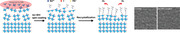

## 1 Introduction

Tin‐based halide perovskites have emerged as promising alternatives to conventional lead‐based halide perovskites. They possess suitable bandgaps, potentially better optoelectronic properties, and most importantly, lower toxicity.^[^ ^1^ ^]^ However, despite sharing a similar ionic structure with Sn^2+^ and Pb^2+^ ions, the record power conversion efficiencies (PCEs) of the tin‐based PSCs currently lag far behind their lead analogs (14.8%^[^ ^2^ ^]^ vs 26.1%^[^ ^3^ ^]^). This performance gap can be attributed to the active 5*s* electrons of Sn^2+^ ions, which induce several challenges in achieving efficient PSCs, including uncontrollable and rapid crystallization processes,^[^ ^4^ ^]^ poor film coverage,^[^ ^5^ ^]^ vulnerability to oxidation,^[^ ^6^ ^]^ and the formation of defective crystals.^[^ ^7^ ^]^ In contrast, Pb^2+^ ions effectively retain their outermost 6*s* electrons due to the stronger inert‐pair effect, resisting further oxidation.^[^ ^8^ ^]^

Studies have revealed that the surface of tin‐based perovskite films is particularly prone to oxidation compared to the bulk film, due to the valance band surface pinning effect.^[^ ^9^, ^10^ ^]^ Oxidation at the surface can propagate into the bulk film by dissociating the weak Sn─I bonds, resulting in further deterioration of film quality.^[^ ^6^, ^11^ ^]^ The flawed surface possesses numerous non‐radiative recombination sites and unregulated strain,^[^ ^12^, ^13^ ^]^ which hinder charge transport and diminish device efficiency.^[^ ^10^ ^]^

Numerous surface treatment strategies have been developed for lead‐based perovskite systems. For instance, various long‐chain ammonium ligands have been employed to passivate the perovskite surfaces, improving both device performance and stability by acting as oxygen and moisture barriers and passivating interfaces.^[^ ^14^, ^15^ ^]^ Butylammonium ions have been widely reported as effective ligands by passivating the grain boundaries^[^ ^16^ ^]^ or constructing a protective 2D perovskite layer,^[^ ^14^ ^]^ with common solvents, such as isopropyl alcohol (IPA). However, applying similar surface treatments to tin‐based systems has not yielded the same success. One reason for this disparity is the high solubility of tin‐based perovskites in common solvents like IPA,^[^ ^17^, ^18^ ^]^ which can destroy the photo‐active perovskite layer, posing practical challenges for depositing treatment agents. Seok et al. attempted to passivate the surface of tin‐based perovskite with thiophenemethylammonium iodine(ThMAI) but failed to achieve a promising PCE mainly due to the usage of IPA.^[^ ^19^ ^]^ Diau et al. introduced hexafluoro‐2‐propanol as the solvent for various ligands, which has relatively lower solubility than IPA, observed the formation of 2D/3D perovskite structure, which achieved over 10% PCE with improved stability.^[^ ^18^ ^]^ It is suggested that there is a pressing demand for the development and comprehension of tailored surface treatment strategies specifically designed for tin‐based perovskites.

In this work, we introduce an effective surface treatment strategy using an *iso*‐butylammonium iodide (*iso*‐BAI) solution to improve the perovskite film surface quality and the corresponding device performance. A mixed solvent composition of 5% 2‐methyl‐2‐butanol (MB) and 95% chlorobenzene (CB) were employed, which exhibits moderate solubility to dissolve the defective surface while preserving the bulk. Despite *iso*‐BA^+^ being a commonly employed A’‐site cation for forming 2D perovskites,^[^ ^20^, ^21^, ^22^ ^]^ this treatment did not produce detectable 2D perovskite signals. Instead, depth profiling of the perovskite film confirmed a significant enhancement in crystallinity at the perovskite surface, with the bulk structure remaining unaffected after the treatment. We conducted in situ measurements to delve into the dissolution‐recrystallization process at the surface. The results suggested that *iso*‐BA^+^ ions act as anchoring sites to guide the recrystallization, resulting in a substantial enhancement in crystallinity. Moreover, this controlled recrystallization notably alleviated surface strain. After the treatment, *iso*‐BAI remained at the surface to prevent direct contact with oxygen and moisture. The surface‐treated perovskite films exhibited enhanced morphology, better optoelectronic properties, and greater resistance to oxidation. Consequently, the PCE of the devices was boosted from 11.8% to 14.2%, nearly approaching the current record. This work presents a facile approach to rectify the defective tin‐based perovskite surface. It also contributes to a deeper understanding of surface treatment mechanisms in tin‐based perovskite systems, which can significantly differ from those of lead‐based counterparts, offering valuable insights for customized strategies to improve the performance of tin‐based perovskites.

### 1.1 Surface Characterization of Tin‐Based Perovskite Films

**Figure 1:**
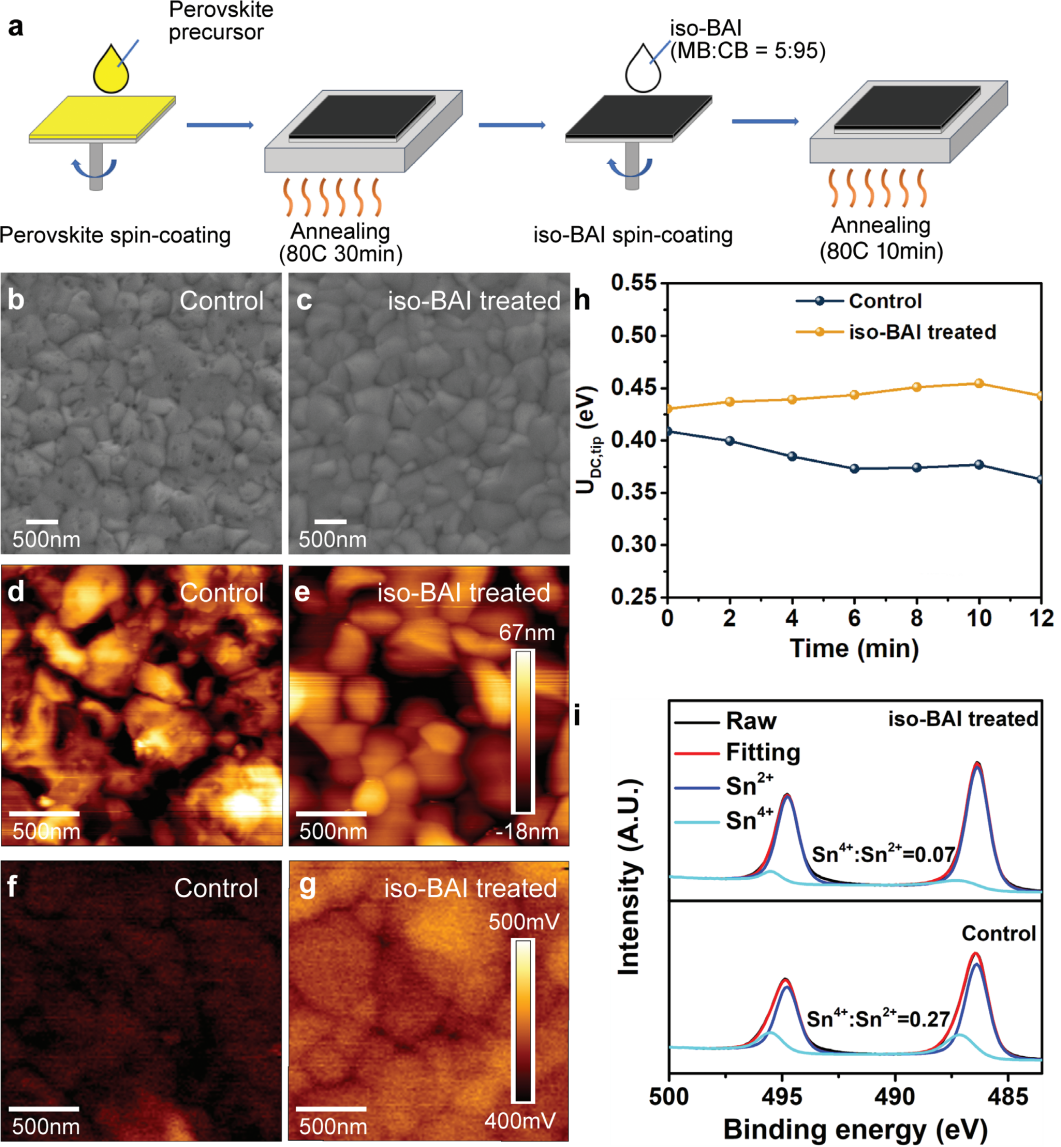
a) Schematics of the fabrication procedure of FASnI_3_ perovskite thin films with the *iso*‐BAI treatment. SEM images of the b) control and c) *iso*‐BAI treated FASnI_3_ perovskite films. The corresponding d,e) AFM and f,g) KPFM images. The scanning area is 2 µm × 2 µm. (h) Time evolution of the averaged *U*_DC,tip_ over the entire scanning area of both films(i) Sn 3d XPS spectra of both films.

As illustrated in **Figure** **1a**, FASnI_3_ perovskite films were prepared through a one‐step spin‐coating and annealing process,^[^ ^13^ ^]^ followed by treatment with the *iso*‐BAI solvent via spin‐coating and subsequent annealing. The detailed fabrication process is provided in the Method section. A mixed solvent containing chlorobenzene (CB) and 2‐methyl‐2‐butanol (MB) was used to dissolve *iso*‐BAI. It is worth noting that CB^[^ ^23^ ^]^ is a well‐known anti‐solvent for perovskites, while MB, with relatively bulkier alkyl chains, exhibits mild solubility^[^ ^24^ ^]^ (Figure S1, Supporting Information). The optimal CB:MB ratio was determined to be 95:5, as it yielded a film with the highest crystallinity, as indicated in Figure S2 (Supporting Information).

To gain insights into the impact of the *iso*‐BAI treatment on the perovskite surface morphology, scanning electron microscopy (SEM) measurements were conducted. The control perovskite film exhibits dense pinholes (Figure 1b), which were remarkably eliminated with the *iso*‐BAI treatment (Figure S2, Supporting Information). However, the *iso*‐BAI treated as‐cast film displayed an inhomogeneous surface with micron‐size uneven regions (Figure S3, Supporting Information). Subsequent annealing of the as‐cast film resulted in a pinhole‐free morphology and ameliorated perovskite crystal grains (Figure 1c), demonstrating the essential role of annealing in the *iso*‐BAI treatment. Further investigation of the surface topography was conducted using atomic force microscopy (AFM). Notably, the AFM measurements were performed under ambient air, highlighting the *iso*‐BAI treatment's protective effect against oxidation. The control film showed defective grains with a rough surface, even within individual grains (Figure 1d). In contrast, after the *iso*‐BAI treatment, the grain surfaces become smoother and more compact (Figure 1e). The root‐mean‐square (RMS) of the perovskite surface roughness also decreases from 19.3 to 14.7 nm after the *iso*‐BAI treatment, which is advantageous for subsequent carrier transport layer deposition.

Kelvin probe force microscopy (KPFM) was then employed to investigate potential modifications induced by the *iso*‐BAI treatment on the work function. The compensation bias of the contact potential difference (CPD) (*U*_DC,tip_ =   − *U*_CPD_) was measured. A larger *U*_DC,tip_ corresponds to a lower work function of the sample. The *U*_DC,tip_ recorded with KPFM (Figure 1f,g) was averaged over a scanning area of 2 µm × 2 µm to minimize variations among grains. The averaged *U*_DC,tip_ of the control film (408 meV) was lower than that of the *iso*‐BAI treated film (430 meV), indicating that the iso‐BAI treatment led to a relatively lower work function of the perovskite film. With similar band structures, a higher work function of the control film implies that this Fermi level is closer to the valence band maximum (VBM), signifying a more p‐doped characteristic. This can be attributed to Sn vacancies and oxidation.^[^ ^25^ ^]^ Moreover, after 12 minutes of exposure in air, the *U*_DC,tip_ of the *iso*‐BAI treated film remained almost unchanged whereas the *U*_DC,tip_ of the control film drops by 46 meV (Figure 1h). This drop indicated a trend of further p‐doping caused by tin oxidation. This observation was further corroborated by X‐ray photoelectron spectroscopy (XPS) results, which showed that the control film had a higher Sn^4+^ content (Sn^4+^:Sn^2+^ = 0.27) compared to the much lower Sn^4+^ content in the *iso*‐BAI treated film (Sn^4+^:Sn^2+^ = 0.07), as depicted in Figure 1i. Additionally, XPS spectra taken after sputtering off the surface of the perovskite films (Figure S4, Supporting Information) showed minimal signal from Sn^4+^ for both films, suggesting that the bulk was much less vulnerable to oxidation compared to the surface. All the surface characterizations outlined above collectively demonstrate that the *iso*‐BAI treatment effectively suppress p‐type doping by reducing film surface defects and shielding the film from oxidation.

### 1.2 Film Crystallinity and Strain Analysis

**Figure 2:**
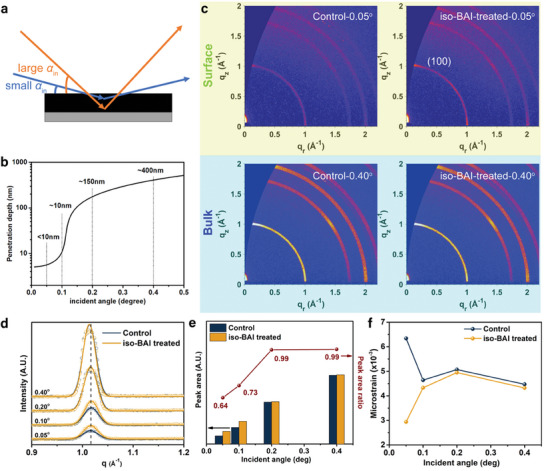
a) Schematics of the GIWAXS measurement with different incident angles. b) The relationship between X‐ray penetration depth and incident angle. c) GIWAXS intensity profiles of the control film and *iso*‐BAI treated film measured at different incident angles. d) GIWAXS intensity profiles (azimuthal integral of the diffraction ring) of the control and the *iso*‐BAI treated films at different incident angles. e) Comparison between the (100) peak areas and the peak area ratio between the control and iso‐BAI treated films at different incident angles. f) Estimated microstrain of the control and *iso*‐BAI treated perovskite films based on Williamson‐Hall analysis.

To examine the influence of the *iso*‐BAI treatment on perovskite crystallites both at the surface and in the bulk, GIWAXS depth profiling was performed by adjusting the incident angle to obtain different penetration depths of X‐rays (**Figure** **2a**). A smaller incident angle provides lattice information from the thinner top layer of the film while a larger incident angle offers the statistical average information from the surface to the penetrated bulk film (Figure 2b).^[^ ^26^ ^]^ The GIWAXS patterns of the perovskite films, without and with the *iso*‐BAI treatment, were measured at various incident angles (0.05°, 0.10°, 0.20°, and 0.40°), as displayed in Figure 2c and Figure S5 (Supporting Information). Interestingly, in contrast to Pb‐based perovskites,^[^ ^14^, ^18^ ^]^ the *iso*‐BAI treatment on Sn‐based perovskites does not result in the transformation of the perovskite surface into 2D perovskites, as suggested by the absence of additional (0k0) peaks after the *iso*‐BAI treatment (Figures S5 and S6, Supporting Information). This is consistent with our recent findings that highly oriented 2D phases is more difficult to form in tin‐based perovskites than lead‐based counterparts, because of the different chemical bonding nature.^[^ ^27^ ^]^ Instead of forming 2D phases, the crystallinity of the *iso*‐BAI treated film surface was significantly enhanced, as evidenced by the larger peak area of the (100) peak at *q*  =  1.01 Å^−1^ obtained at incident angles of 0.05° and 0.10° (Figure 2d). In contrast, at large incident angles of 0.20° and 0.40°, the peak area of both control and treated films converged to a similar level. The ratio between the peak area of the control and the treated film increased from 0.64 at an incident angle of 0.05° to 0.99 at an incident angle of 0.20°, remaining close to unity for larger incident angles (Figure 2e). This result suggests that the *iso*‐BAI treatment mainly modifies the surface of the perovskite film by enhancing crystallinity while preserving the integrity of the bulk, which is beneficial for enhancing charge transport and reducing non‐radiative recombination.

In addition to film crystallinity, the local inhomogeneous microstrain, which is signified by deviations in the *d*‐spacing from the ideal lattice constant, can be estimated using Williamson–Hall analysis^[^ ^28^ ^]^ based on the diffraction peak widths at different diffraction angles with the following formula:

(1) $$\beta \;\cos\theta =\frac{k\lambda }{D}+4\varepsilon \sin\theta$$

where β is the full‐width‐half‐maximum (FWHM) of the diffraction peak, θ is the diffraction angle, *D* is the average crystallite size, and ε is the microstrain. Hence, the slope of the plot with βcos θ against 4sin θ corresponds to the microstrain of the sample. Fitting results for both the control and *iso*‐BAI treated films are summarized in Figure 2f and Figure S7 (Supporting Information). The microstrain of the control film measured at a small incident angle of 0.05° (6.34 × 10^−3^) is much higher than that of the *iso*‐BAI treated film (2.94 × 10^−3^). As the incident angle gradually increases for a deeper probing depth, the microstrain of the control film decreases and eventually reaches a level similar to that of the *iso*‐BAI treated film. These results indicate that the control film experiences more pronounced local lattice deviations at the surface compared to the *iso*‐BAI treated film, in line with the observed more defective surface vulnerable to oxidation of the control film. Intriguingly, the surface of the *iso*‐BAI treated film exhibits even smaller microstrain than the bulk, implying that *iso*‐BAI may play a vital role in the surface reconstruction. This aspect will be discussed in greater detail in the next section.

### 1.3 Mechanisms of the *iso*‐BAI treatment

**Figure 3:**
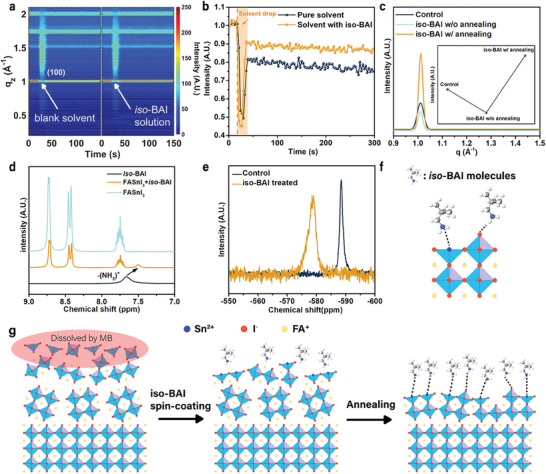
a) In situ GIWAXS measurements of spin‐coating pure solvent or *iso*‐BAI solution on the FASnI_3_ perovskite films. b) Evolution of the normalized peak intensity of the (100) peaks from the in situ GIWAXS measurements. c) Ex situ GIWAXS intensity profiles of the perovskite control film and the perovskite films with *iso*‐BAI treatment before and after the thermal annealing process at an incident angle of 0.05°. d) 1‐H NMR spectra of the *iso*‐BAI, FASnI_3_ + *iso*‐BAI, FASnI_3_ solution in deuterated‐DMSO. e) 119‐Sn NMR spectra of perovskite films without and with *iso*‐BAI treatment. f) Schematics of the interactions between the *iso*‐BAI molecules and the tin‐based perovskite. g) Schematics of perovskite surface dissolution‐recrystallization process with the *iso*‐BAI treatment.

To understand the role of iso‐BAI in the surface treatment, in situ GIWAXS measurements were carried out to monitor the FASnI_3_ film dissolution‐recrystallization process when spin‐coating the pristine solvent (CB:MB = 95:5) or the *iso*‐BAI solution on top (**Figure** **3**a). Given the absence of secondary phase peaks, we mainly focused on the intensity evolution of the (100) perovskite peak at *q*  =  1.01 Å^−1^ to track changes in film crystallinity (Figure 3b). For the pristine solvent, the (100) peak intensity decreased to ≈80% of the initial value, demonstrating the moderate solubility of the solvent. Note that the significant drop during the solvent dripping (5th s – 12th s) resulted from X‐ray blockage by the solvent (marked as the pale‐yellow window in Figure 3b). The peak intensity after spin‐coating the *iso*‐BAI also decreased to ≈90%, implying that the presence of *iso*‐BAI could reduce the dissolution of the film to some extent. Remarkably, the subsequent annealing process can significantly enhance the crystallinity of the *iso*‐BAI treated film, surpassing that of the control film, while the pristine solvent cannot (Figure 3c; Figure S10, Supporting Information). This observation underscores the crucial role of *iso*‐BAI in facilitating the recrystallization of the perovskite surface.

To further elucidate the underlying mechanism, mass spectroscopy was carried out, which confirmed the existence of *iso*‐BAI in the treated film by the presence of a peak at *m*/*z*  =  74.096 (Figure S8, Supporting Information). We then employed 1‐H and 119‐Sn nuclear magnetic resonance (NMR) to probe the potential interactions between *iso*‐BAI molecules and tin‐based perovskites, as well as changes in the chemical environment of tin ions, respectively. The 1‐H NMR spectra exhibited a chemical shift of the hydrogen protons corresponding to the ─(NH_3_)^+^ group (Figure 3d). This peak experienced an upfield chemical shift after the treatment, indicating the existence of Lewis‐base interaction between the two species, as previously reported.^[^ ^29^ ^]^ While the electron‐accepting hydrogen bonded with iodide ions, the electron rich‐nitrogen might complement the undercoordinated Sn^2+^. The 119‐Sn NMR results (Figure 3e) further confirmed the change of chemical environment of Sn by a peak shift from −579 to −588 ppm.^[^ ^30^ ^]^ Previous reports suggested that the lone pair electrons of N‐H could enrich the electron density near Sn^2+^ ions, thus improving air stability.^[^ ^31^ ^]^ Both NMR results indicated that *iso*‐BAI molecules interacted with tin perovskites (Figure 3f), explaining their active role in coordinating the perovskite in the re‐crystallization process.

Based on the above result, we propose the working mechanism of the *iso*‐BAI treatment in Figure 3g. Previous simulations have suggested the exceptional vulnerability of the surface of tin‐based perovskites to tin(II) oxidation, serving as the initiation point for film degradation.^[^ ^6^, ^9^ ^]^ Consequently, the iso‐BAI solution, designed with moderate solubility to perovskites, first dissolves the top disordered and defective surface layer, and then *iso*‐BAI molecules act as anchoring sites and form bonds with dissolved Sn^2+^ ions, preventing their removal while facilitating their recrystallization into perovskites.

### 1.4 Optoelectronic Properties and Device Performance

**Figure 4:**
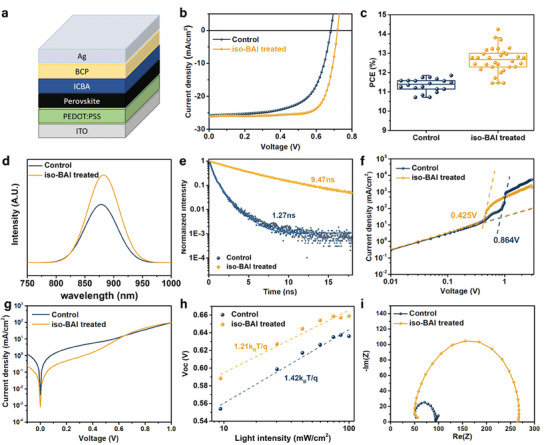
a) Device structure of the FASnI_3_‐based PSCs (ITO/PEDOT:PSS/FASnI_3_/ICBA/BCP/Ag). b) *J*–*V* curves of the best performing devices and c) Efficiency statistics of PSCs based on FASnI_3_ films without and with the *iso*‐BAI treatment. The corresponding d) steady‐state photoluminescence spectra, e) time‐resolved photoluminescence spectra, f) space‐charge‐limited‐current analysis, g) dark *J–V*, h) Light dependence of *V*_oc_, and i) electrochemical impedance spectroscopy results.

To investigate the effect of the *iso*‐BAI treatment on device performance, PSCs were fabricated with the inverted structure (*p–i–n*), as illustrated in **Figure** **4**a. The best‐performing device efficiencies of PSCs exhibited a notable improvement, increasing from 11.8% without the treatment to 14.2% after the *iso*‐BAI treatment (Figure 4b). Further details on device statistics are summarized in Figure S9 (Supporting Information). The increase in efficiency was manifested by improvements in *V*oc (0.681 to 0.719 V), *J*sc, (25.8 to 26.1 mA cm^−2^) and, notably, *FF* (64.1% to 75.7%). Both the devices treated with the pristine solvent and with *iso*‐BAI but without annealing showed poor device performance, as expected (Figure S10, Supporting Information). The performance of devices fabricated with *iso*‐BAI treatment in different solvent compositions, with various solubility, are shown in Figure S11 (Supporting Information). The results suggest that an optimal solubility is desirable, while pure IPA severely damaged the perovskite film, leading to a nearly zero device PCE. Steady‐state photoluminescence (Figure 4d) showed a stronger emission peak at ≈880 m for the *iso*‐BAI treated film, indicating considerably suppressed non‐radiative recombination. Time‐resolved photoluminescence (TRPL) spectra (Figure 4e) of the *iso*‐BAI treated film revealed a significantly longer lifetime (9.47 ns) compared to the control film (1.27 ns). The correspondingly photoluminescence quantum yield (PLQY) also nearly tripled from 4.16% to 11.87% after the iso‐BAI treatment, in line with the *V*_oc_ improvement (Figure S12, Supporting Information). Space‐charge‐limited‐current (SCLC) measurements were used to assess trap density in the films via the so‐called “trap‐filled‐limit voltage” formula^[^ ^32^ ^]^:

(2) $$V_{\mathrm{tfl}}=\frac{qn_{t}L^{2}}{2\varepsilon }$$

where *q* is the elementary charge, *n*_t_ is the trap density, *L* is the thickness of the film and ε is the dielectric constant. As shown in Figure 4f, the *V*_tfl_ of the *iso*‐BAI treated film is fitted to be 0.425 V, much lower than 0.854 V for the control film. The corresponding trap density of the control and the *iso*‐BAI treated devices are calculated to be 3.97 × 10^16^cm^−3^ and 1.96 × 10^16^cm^−3^, respectively, indicating a lower trap density in the *iso*‐BAI treated film. The absorption spectra and corresponding optical band gaps obtained from Tauc plots were similar for both films (Figure S13, Supporting Information). The dark current in the *iso*‐BAI treated device (8.2 × 10^−4^mA cm^−2^) was also noticeably smaller than the control device(4.3 × 10^−3^mA cm^−2^), further suggesting the suppression of deep traps and leakage(Figure 4g). Ideality factors of both devices were obtained by fitting against the plot of *V*_oc_ versus light intensity (log)^[^ ^33^ ^]^ (Figure 4h). The ideality factor of the iso‐BAI treated device (n = 1.21) is smaller than the control device (n = 1.42), indicating suppressed trap‐assisted charge recombination. Electrochemical impedance spectroscopy (EIS) was also carried out to study the recombination status of both films (Figure 4i, Figure S12, Supporting Information). The Nyquist plots fitted with a single RC circuit, as shown in the inset of Figure S14 (Supporting Information), revealed that the recombination resistance of the control film (49.1 Ω) is much smaller than that of the *iso*‐BAI treated film (94.4 Ω), further suggesting that the *iso*‐BAI treatment can effectively suppress the recombination. The storage stability in a nitrogen‐filled glovebox of the *iso*‐BAI treated devices also slightly outperforms the control devices, maintaining 60% of the original PCE (Figure S15, Supporting Information). The limited improvement shown may be related to the relatively high humidity of the glovebox (H_2_O > 1 ppm, O_2_ < 0.01 ppm) (Supplementary Note S1, Supporting Information). Overall, these measurements demonstrated better optoelectronic properties of the *iso*‐BAI treated film, consistent with the observed surface quality after the treatment.

## 2 Conclusion

In summary, we report a straightforward and effective surface treatment strategy that employs the *iso*‐BAI solution to rectify the defective surface of tin‐based perovskite. Importantly, the underlying mechanism differs significantly from a similar treatment on lead‐based systems, where a hydrophobic 2D perovskite was formed on the surface to passivate the surface defects and resist bulk perovskite degradation.^[^ ^14^ ^]^ In our case, the relatively vulnerable tin‐based perovskite surface was initially dissolved by spin‐coating the iso‐BAI solution on top and then recrystallized during the subsequent annealing. The resulting film displayed a substantial improvement in crystallinity and reduction in surface strain, pinholes and roughness, while preserving an intact bulk. The presence of *iso*‐BAI assists in localizing the dissolved Sn^2+^ ions for orderly recrystallization. Promisingly, the surface rectification leads to significant enhancements in optoelectronic properties, contributing to improved charge transport, reduced recombination, and ultimately achieving a notable PCE of 14.2%. This study underscores the distinct mechanism of the surface treatment in tin‐based perovskite compared to their lead‐based counterparts, emphasizing the need for tailed strategies for tin‐based perovskites rather than direct adoption from lead‐based perovskite systems.

## Conflict of Interest

The authors declare no conflict of interest.

## Supporting information

Supporting Information

## Data Availability

The data that support the findings of this study are available in the supplementary material of this article.
