# Supplementary material for: iso‐BAI Guided Surface Recrystallization for Over 14% Tin Halide Perovskite Solar Cells
Source: Adv Sci (Weinh). 2024 Mar 27;11(22):2309668. doi: 10.1002/advs.202309668 (PMC11165555; doi:10.1002/advs.202309668)
Supplement: Supplementary file 1 — Supporting Information [file ADVS-11-2309668-s001.pdf]

ADVANCED SCIENCE
Open Access

Supporting Information

for *Adv. Sci.*, DOI 10.1002/advs.202309668

iso-BAI Guided Surface Recrystallization for Over 14% Tin Halide Perovskite Solar Cells

Pok Fung Chan, Minchao Qin, Chun-Jen Su, Liping Ye, Xuezhou Wang, Yunfan Wang, Xin Guan, Zhen Lu, Gang Li, To Ngai, Sai Wing Tsang, Ni Zhao and Xinhui Lu**

Supporting Information

iso-BAI Guided Surface Recrystallization for Over 14% Tin Halide Perovskite Solar Cells

Pok Fung Chan[1], Minchao Qin[1,], Chun-Jen Su[2], Liping Ye[3], Xuezhou Wang[4], Yunfan Wang[5], Xin Guan[3], Zhen Lu[6], Gang Li[6], To Ngai[3], Sai Wing Tsang[5], Ni Zhao[4], and Xinhui Lu[1,*]*

[1]Department of Physics, The Chinese University of Hong Kong, New Territories 999077, Hong Kong SAR, China

[2]National Synchrotron Radiation Research Center, Hsinchu Science Park, Hsinchu 30076, Taiwan

[3]Department of Chemistry, The Chinese University of Hong Kong, New Territories 999077, Hong Kong SAR, China

[4]Department of Electronic Engineering, The Chinese University of Hong Kong, New Territories 999077, Hong Kong SAR, China

[5]Department of Materials Science and Engineering, City University of Hong Kong, Kowloon Tong 999077, Hong Kong, China

[6]Department of Electrical and Electronic Engineering, The Hong Kong Polytechnic University, Hung Hom 999077, Hong Kong SAR, China

Experimental Section/Methods

Materials

Formamidinium iodide (FAI), methylammonium iodide (MAI), and iso-Butylammonium iodide were purchased from Greatcell Solar; ITO glass was purchased from Advanced Election Technology Co. Ltd; Poly(3,4-ethylenedioxythiophene) polystyrene sulfonate (PEDOT:PSS, Al 4083) was purchased from Ossila Ltd;. Indene-

C60 Bisadduct (ICBA) was purchased from 1-Material Inc. (6,6)-Phenyl-C_{61}-butyric acid methyl ester (PCBM) was purchased from Xi'an Polymer Light Technology Corp. All the other chemical materials were purchased from Sigma-Aldrich and used as received unless stated otherwise.

Device fabrication

ITO substrates were sequentially rinsed by sonication in detergent, deionized (DI) water, acetone, and isopropanol for 30 min, respectively, and then dried under nitrogen gas before use. Cleaned ITO substrates were treated with ultraviolet-ozone for 15 min, followed by the deposition of a hole transporting layer of PEDOT:PSS by spin-coating the PEDOT:PSS solution at 4500 rpm for 30 s, and then annealed at 130 ºC for 30 min. The SnI_2 precursor was prepared by dissolving I_2 (1.0 M) in a mixture of DMF/DMSO with the volume ratio of 4:1, and then excess Sn powder was added, followed by a 30min of vigorous shaking. The $FASnI_3$ perovskite precursor was prepared by dissolving FAI (0.95 M), MAI (0.05 M), and SnF_2 (0.1 M) into the SnI_2 precursor, and then excess 6% 1.5 M PEAI was added into the precursor. The perovskite precursor was filtered to isolate the tin powder before use. The *iso*-BAI was prepared by dissolving 1mg/mL *iso*-BAI in 5:95 (volume ratio) MB:CB. The ICBA solution was prepared by dissolving 18mg/mL ICBA in CB. The BCP solution was prepared by dissolving 0.5mg/mL BCP in IPA. The perovskite film was deposited by spin-coating the perovskite precursor on the ITO/PEDOT:PSS substrate at 4500 rpm for 50 s, and 100 uL chlorobenzene containing 0.5 mg/ml PCBM was dripped onto the substrate at the 9th second from the start of spin-coating. The as-cast film was then annealed at 80 ºC for 30 min. For the *iso*-BAI treatment, 50uL was dripped onto the annealed perovskite film at 4000rpm for 20s, followed by an annealing process at 80 ºC for 10min.

ICBA and BCP solutions were subsequently spin-coated at 2500 rpm and 4000 rpm for 20 s, respectively. Finally, a 100 nm Ag electrode was deposited by thermal evaporation.

Characterizations

The crystalline structures for the perovskite films were measured by XRD on a Rigaku Smart Lab (λ = 1.54 Å). GIWAXS measurements were carried out using a Xeuss 2.0 SAXS/WAXS laboratory beamline with a Cu X-ray source (8.05 keV, 1.54 Å) and a Pilatus 3R 300K detector. In situ GIWAXS experiments for the perovskite spin-coating process and light stability tests were conducted at TLS 23A small- and wide-angle X-ray scattering (SWAXS) beamline at the National Synchrotron Radiation Research Center (NSRRC), Hsinchu, Taiwan. The *J-V* curves were measured by a Keysight B2901A source meter unit under an AM 1.5G solar simulator (SS-F5; Enli Technology, Taiwan), and the light intensity was calibrated using a standard silicon reference cell. X-ray photoelectron spectroscopy (XPS) characterizations were performed at BL09A2 U5 beamline at the National Synchrotron Radiation Research Centre, Taiwan. The incident photon energy is 750 eV and the data were calibrated using an Au sample. UV-Vis absorption spectra were taken on a Hitachi U-3501 ultraviolet/visible/near-infrared spectrophotometer. The perovskite surface morphology was characterized by a high-resolution field emission scanning electron microscopy (HR-FESEM) (FEI, Quanta 400). Photoluminescence measurements of perovskite films on glass were conducted by using an Edinburgh FLSP920 spectrophotometer installed with an excitation source of 485 nm picosecond pulsed diode laser with an average power of 0.15 mW. Both the ^{1}H-NMR and ^{119}Sn-NMR spectra are recorded with the Brucker AVANCE III 400 NMR, and chemical shift values(δ) are expressed in parts per million using residual solvents protons as internal standard. AFM and KPFM measurements were conducted with the Nikon Ti Inverted

microscope bruker and the JPK atom force microscope with a single photon spectrometer. The probing tip was the ElectriMulti75-G probe from Budget Sensors. Electrochemical impedance spectroscopy (EIS) measurements were conducted using Zahner Electrochemical workstation. The steady-state PL measurement is conducted with the home-made setup by Tsang's group[1]. TRPL measurements are conducted using a photoluminescence spectrometer (iHR320, HORIBA). A 463 nm laser diode (DeltaDiode-470L, Horiba) as a pump source was used for photoexcitation with the frequency of 100 MHz, . The TRPL spectrum was obtained by a high-sensitivity photon counting detector (TRPL-PPD900-MICOS, horiba). PLQY measurements are conducted by mounting the perovskite films in an integrating sphere with Enlitech LQ-100X-PL.

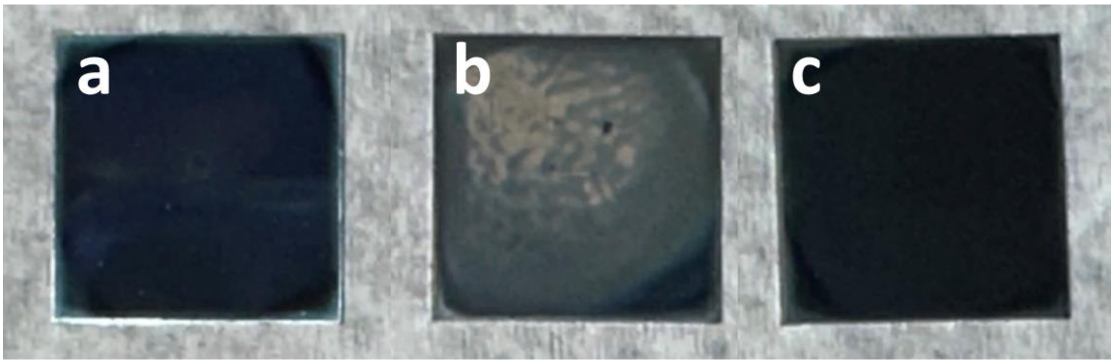

Figure S1. (a) Perovskite film **without** any surface treatment (b) Perovskite film treated with iso-BAI in **IPA** (c) Perovskite film treated with iso-BAI in **MB**.

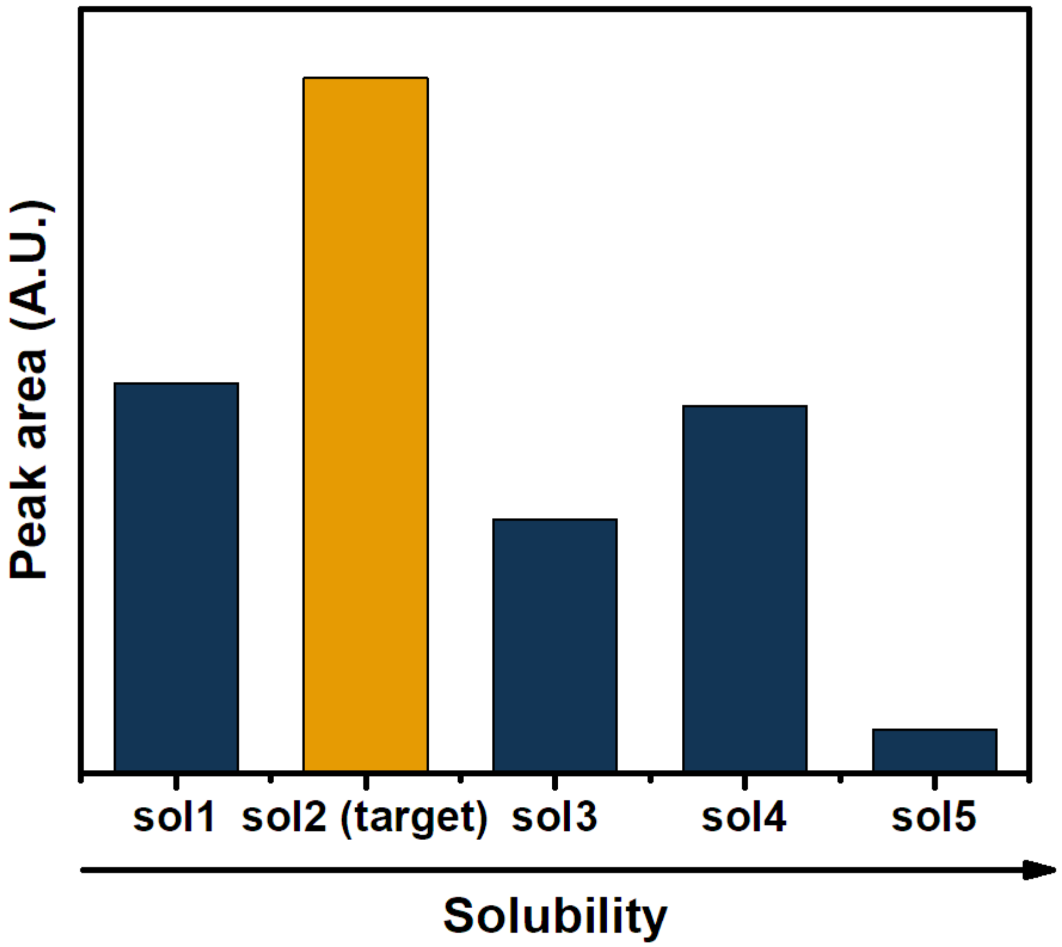

Figure S2. Peak area of the (100) peak of the perovskite films treated with *iso*-BAI different solvent compositions. The solvent compositions are arranged in increasing solubility, namely sol 1,2,3,4,5,6 corresponding to MB:CB(1:99), MB:CB(5:95), pure MB, MB:IPA(80:20), and pure IPA respectively.

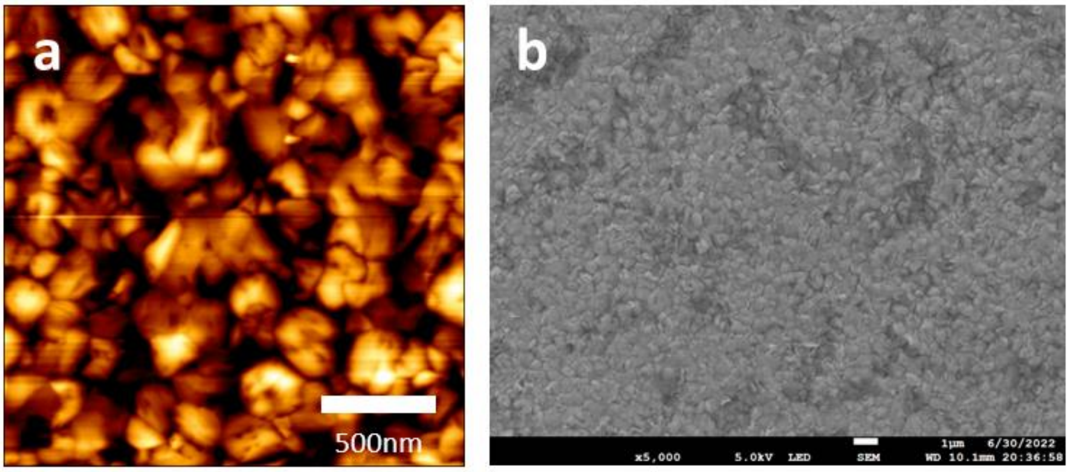

Figure S3. (a) AFM, and (b) SEM images of the iso-BAI treated film **before** annealing.

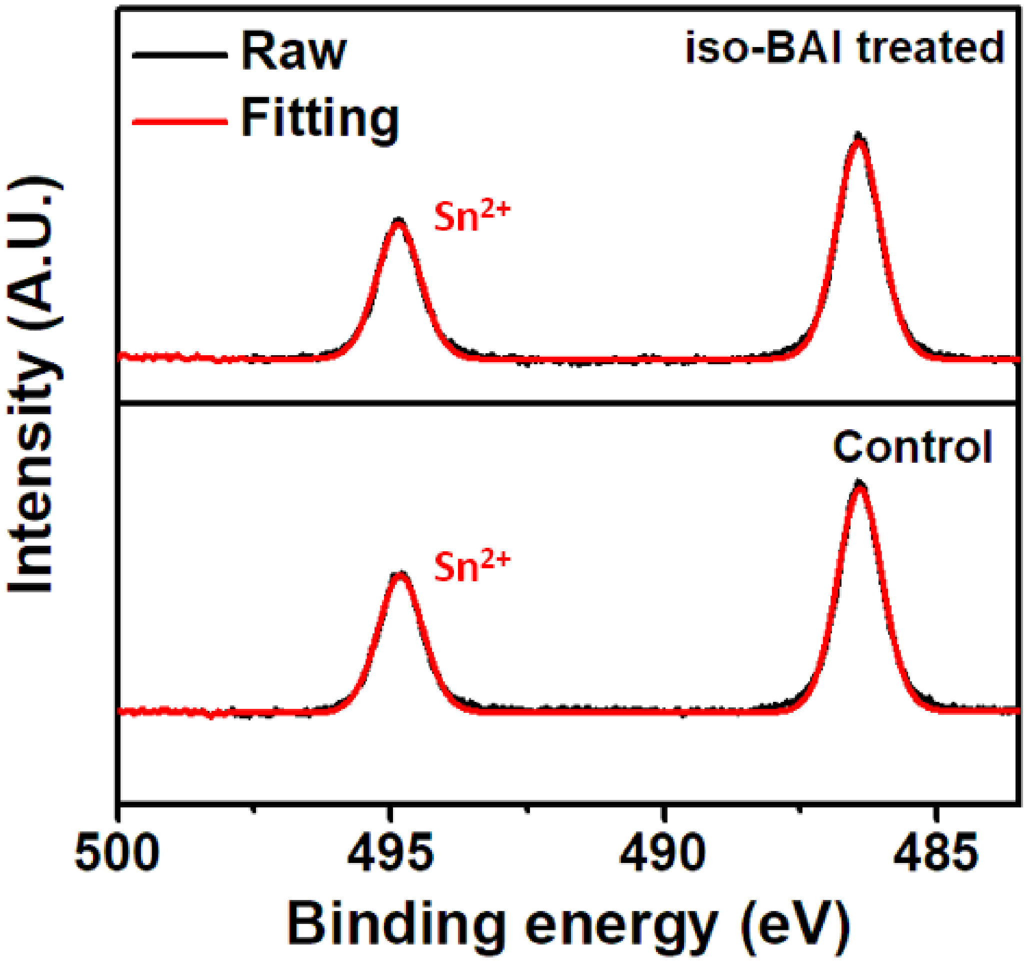

Figure S4. XPS spectra of the perovskite film after etching **with** and **without** the *iso*-BAI surface treatment.

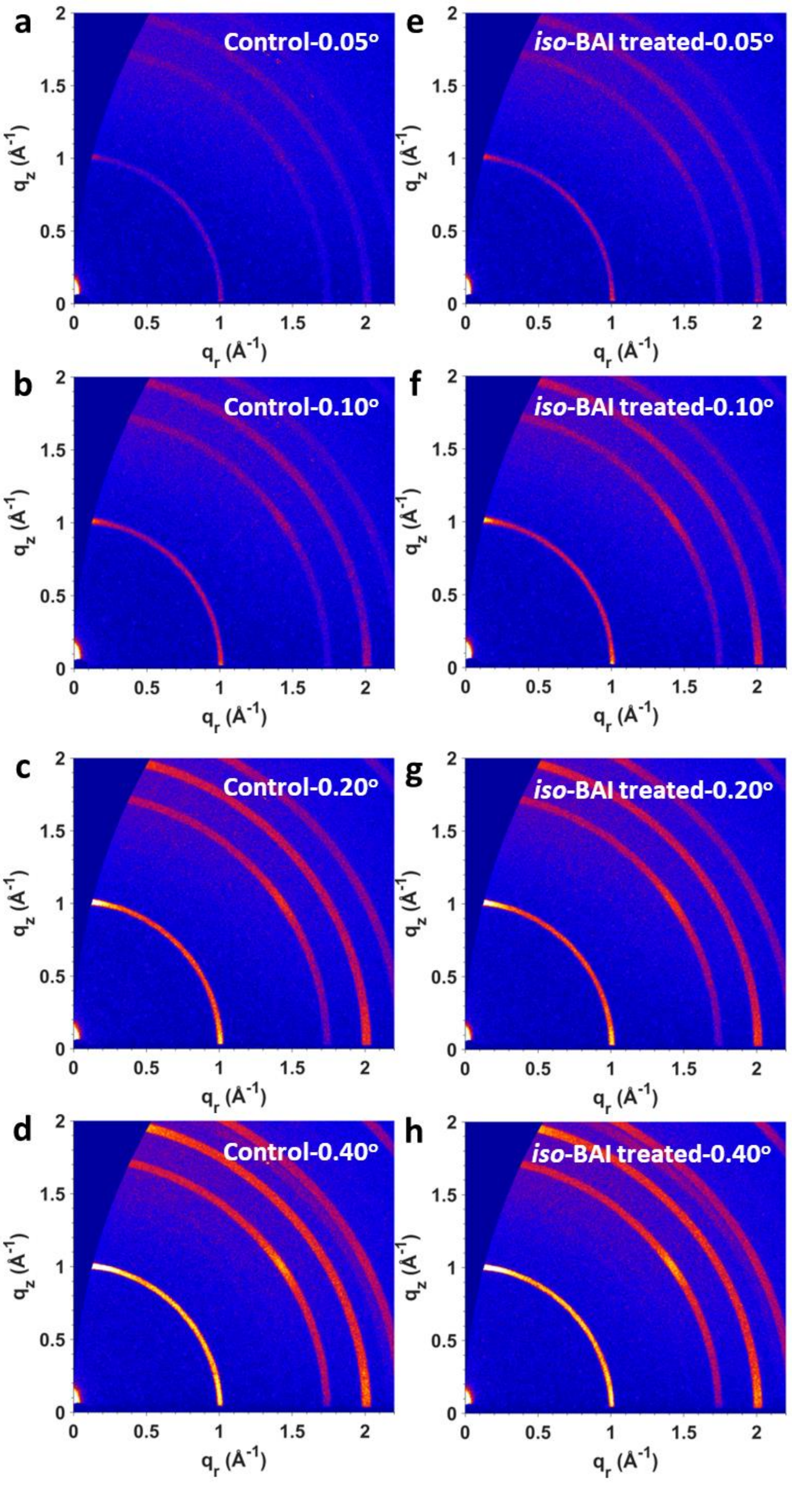

Figure S5. GIWAXS diffraction patterns of the control film (a-d), and the iso-BAI treated film (e-h), at incident angles of 0.05^o, 0.10^o, 0.20^o, 0.40^o.

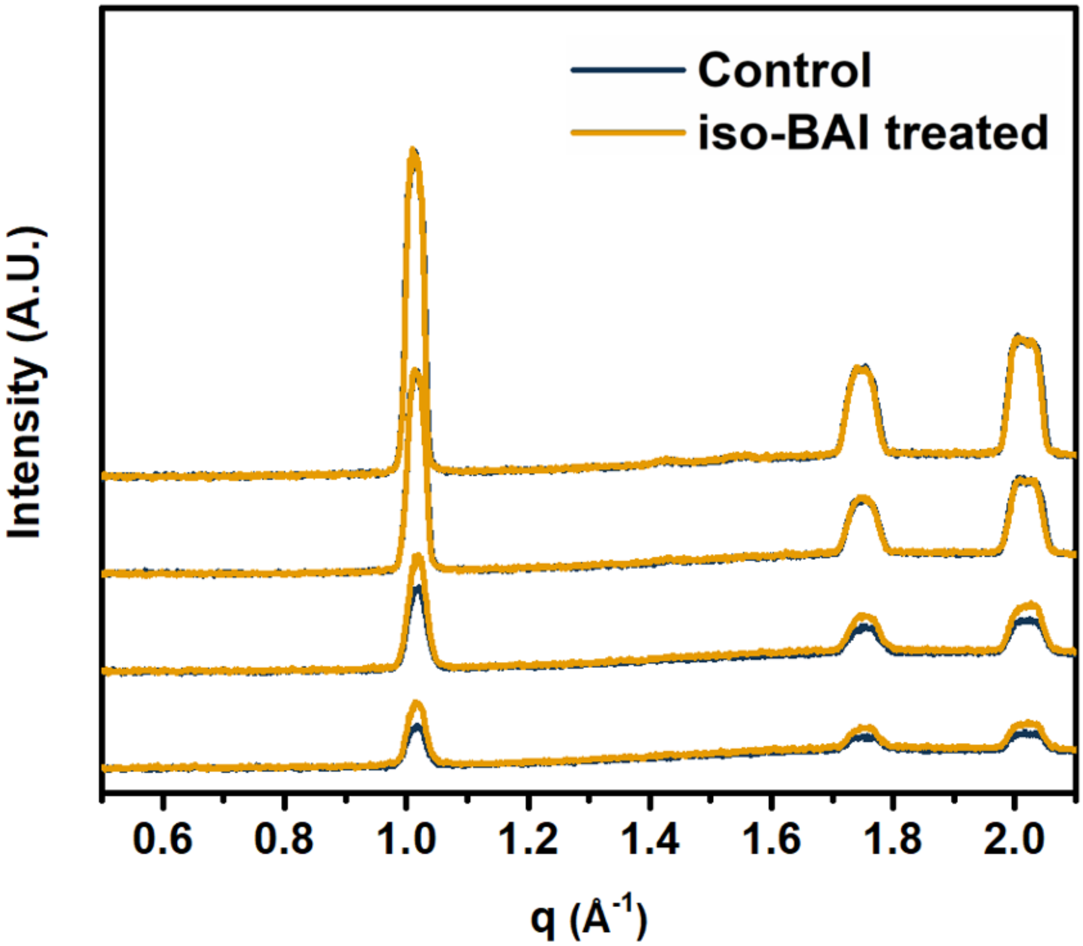

Figure S6. GIWAXS intensity profiles of the control film and *iso*-BAI treated film measured at different incident angles.

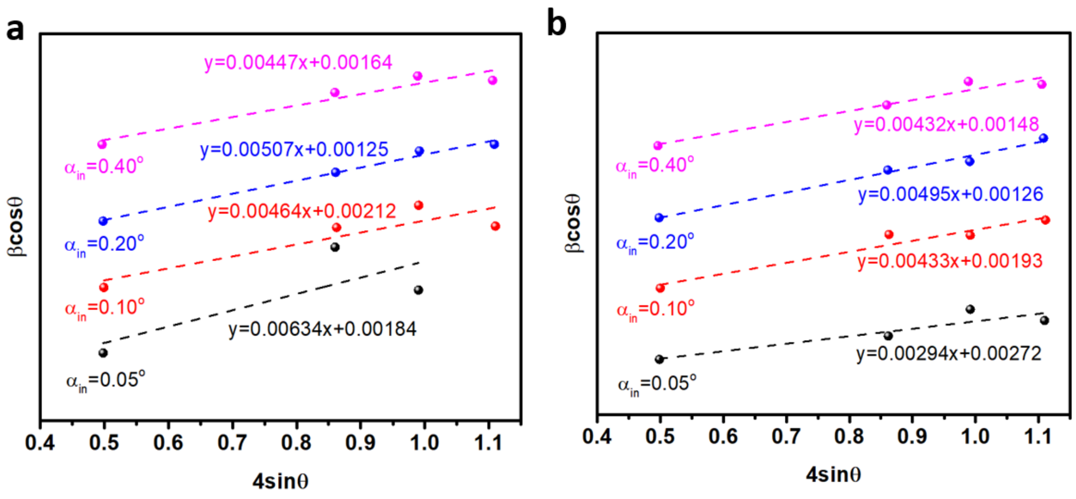

Figure S7. Williamson-Hall analysis of the microstrain of (a) the control film, and (b) the iso-BAI treated film, according to the GIWAXS intensity profiles with different incident angles (0.05°, 0.10°, 0.20°, 0.40°).

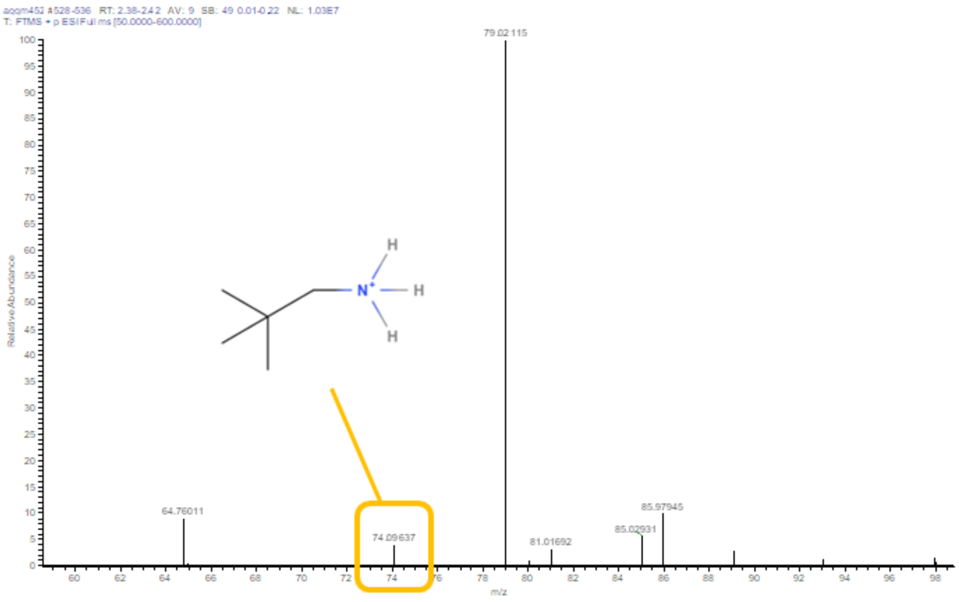

Figure S8. Mass spectroscopy measurement of the iso-BAI treated film. The peak at m/z = 74.096 corresponds to the (CH3)3-CH-CH2-NH3 fragment.

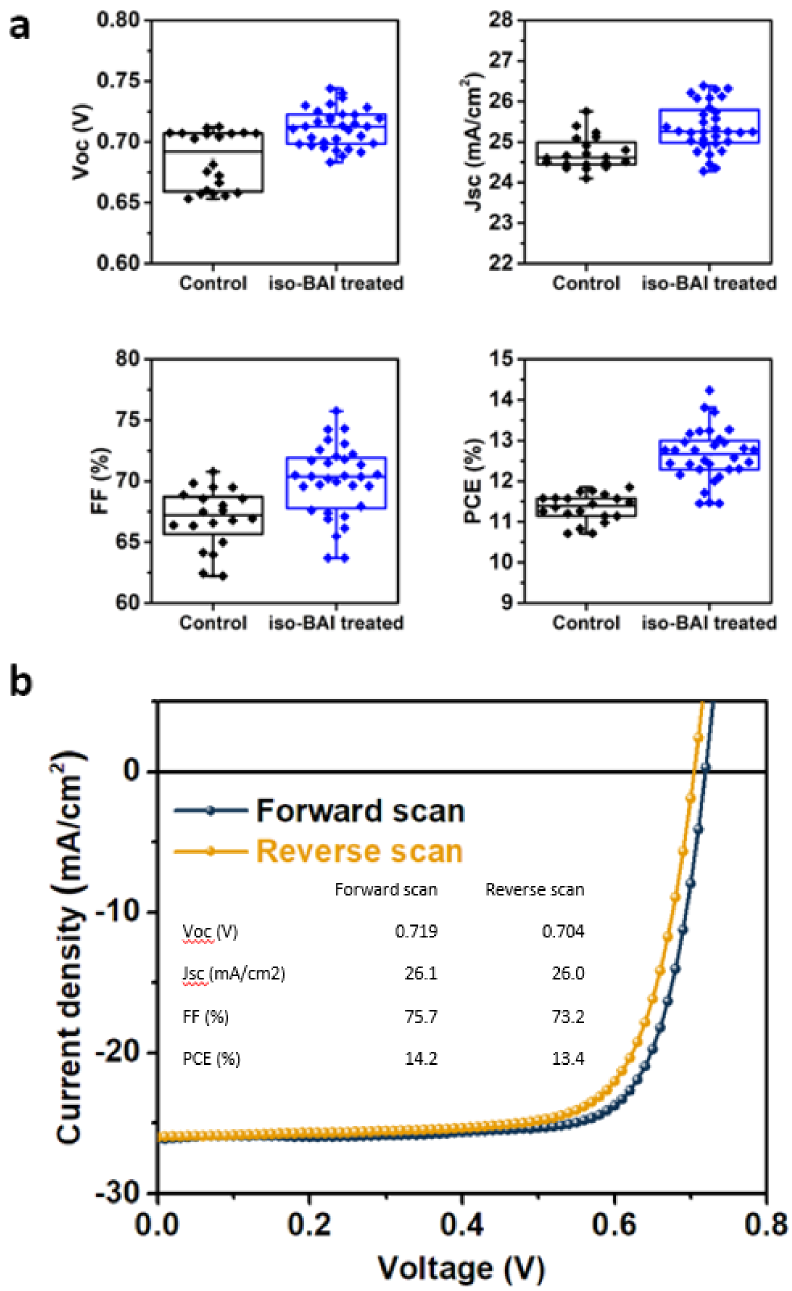

Figure S9. (a) Voc, Jsc, FF, and PCE of the PSC devices fabricated with the control, and the iso-BAI treated film. (b) Forward and reverse scan of the best performing *iso*-BAI treated device.

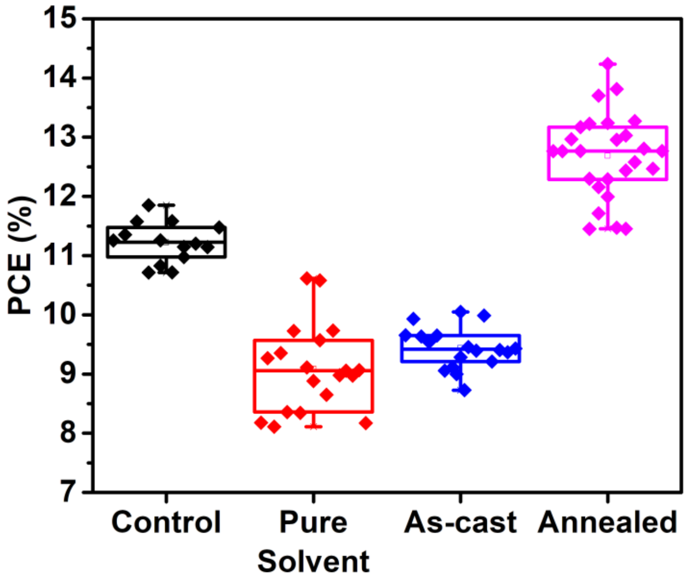

Figure S10. PCE comparison of the PSC devices fabricated with the control film, the pure-solvent-treated film, the iso-BAI treated film **without** annealing, and the iso-BAI treated film **after** annealing.

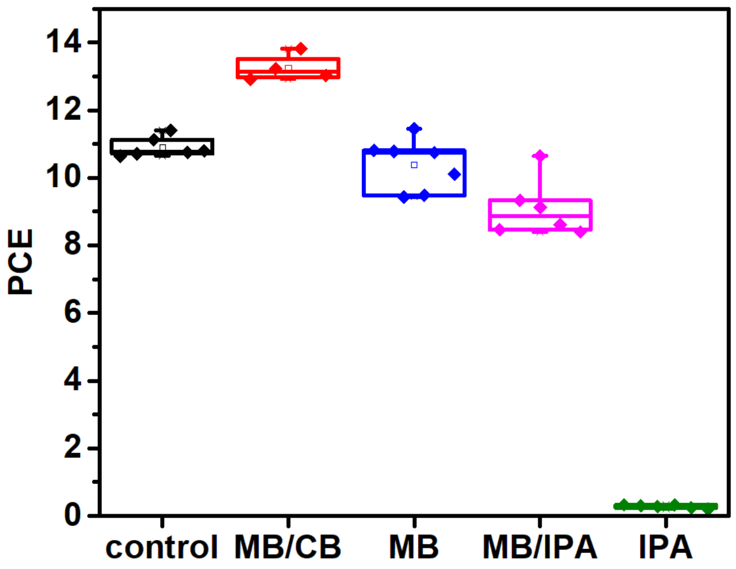

Figure S11. PCE comparison of the PSC devices fabricated with the control film, the *iso*-BAI treated film in the solution of MB/CB (9:95), pure MB, MB/IPA (20:80) and pure IPA.

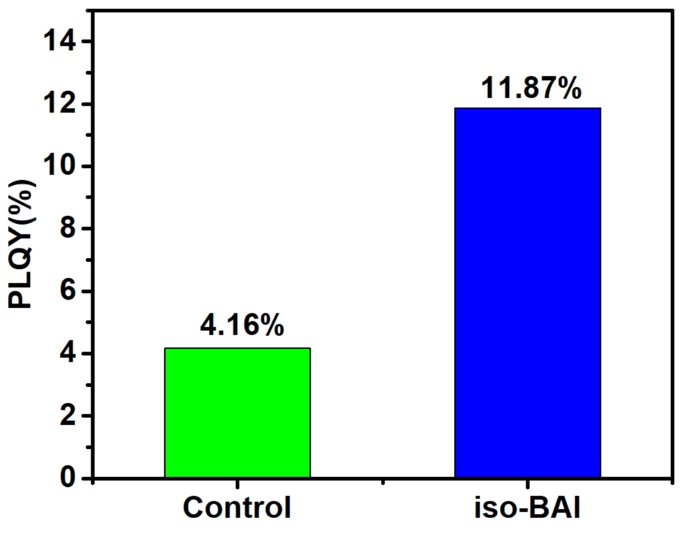

Figure S12. Photoluminescence quantum yield comparison of the control film and the iso-BAI treated film.

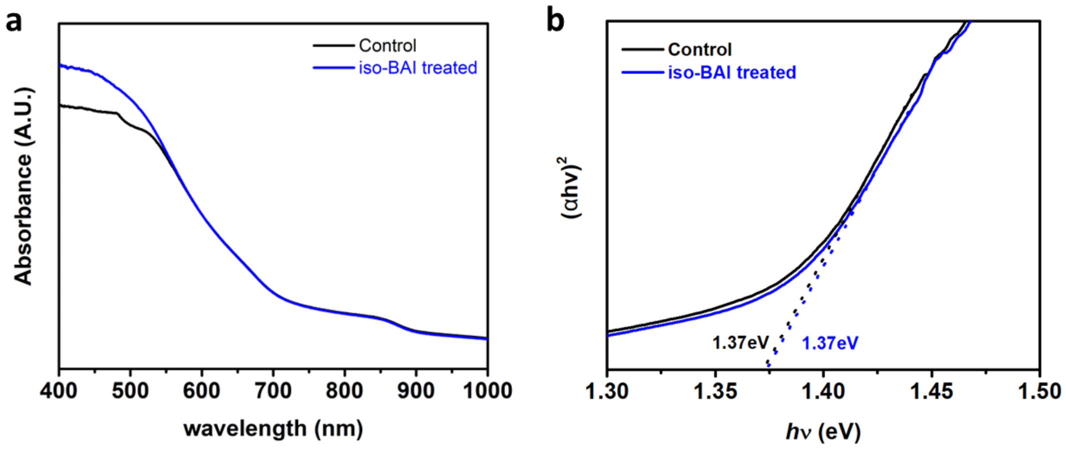

Figure S13. UV-vis spectra of the control film and the iso-BAI treated film, and the corresponding Tauc plot.

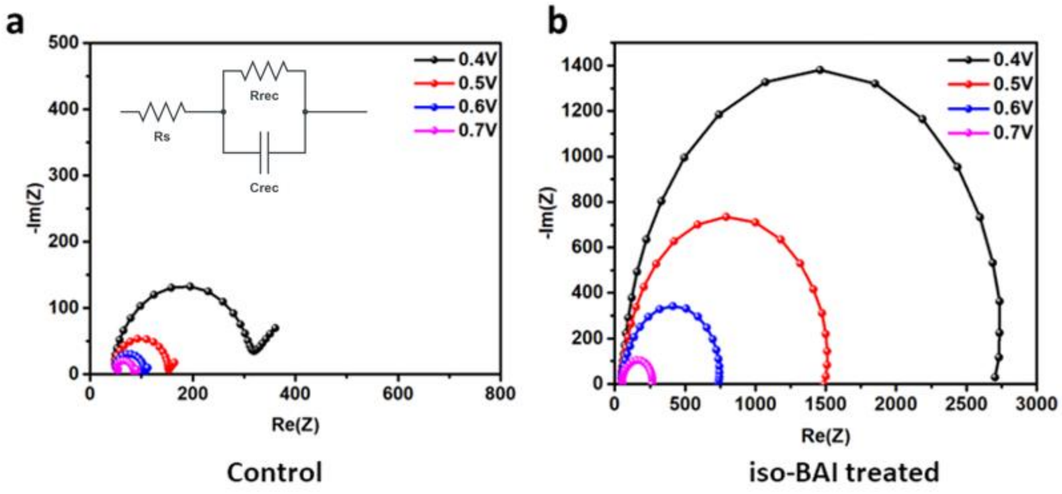

Figure S14. Nyquist plots of the PSC devices fabricated with (a) the control, and (b) the iso-BAI treated film with biases at 0.4V, 0.5V, 0.6V, and 0.7V.

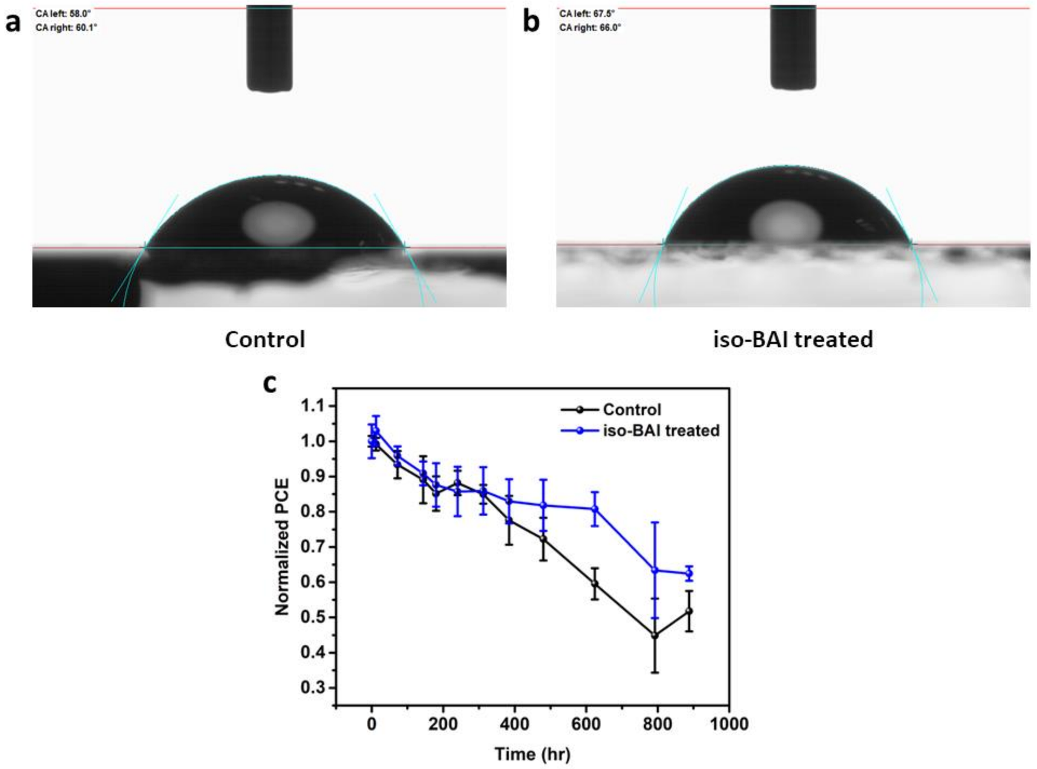

Figure S15. Contact angle measurements with water of (a) the control films, and (b) the iso-BAI treated film. (c) Device stability based on both films under a nitrogen environment for 40 days.

Supplementary Note 1. Regarding the stability of the PSC devices, moisture plays a critical role together with oxygen. Bare perovskite film exposing ambient air, R.H. = 60%, degraded rapidly in several hours. In contrast, a similar storage in a dry box with R.H. = 25%, the perovskite films retained the black phase after one week. The contact angle measurement shown in **Figure S15** indicates that the water resistance with the iso-BAI treatment is limited. This probably restrict the stability improvement.

References

1. Guan, Z., et al., *High‐Efficiency Blue Perovskite Light‐Emitting Diodes with Improved Photoluminescence Quantum Yield via Reducing Trap‐Induced Recombination and Exciton‐Exciton Annihilation.* Advanced Functional Materials, 2022. **32**(40).
